# Seasonal changes in rosemary species: A chemotaxonomic assessment of two varieties based on essential oil compounds, antioxidant and antibacterial activities

**DOI:** 10.1371/journal.pone.0273367

**Published:** 2022-08-29

**Authors:** Abdelkarim Ben Arfa, Hassen Gouja, Hédia Hannachi, Hiroko Isoda, Mohamed Neffati, Hanen Najjaa

**Affiliations:** 1 Laboratoire des Ecosystèmes Pastoraux et de Valorisation des Plantes Spontanées, LR16IRA03, Institut des Régions Arides, Université de Gabès, Medenine, Tunisie; 2 Laboratory of Plant Productivity and Environmental Constraint, LR18ES04, Biology Department, Faculty of Sciences, University Tunis El Manar, Tunis, Tunisia; 3 Alliance for Research on the Mediterranean and North Africa (ARENA), University of Tsukuba, Tsukuba, Japan; Tocklai Tea Research Institute, INDIA

## Abstract

Rosemary (*Rosmarinus officinalis* L.) is a popular herb in cooking, traditional healing, and aromatherapy. This study was conducted to evaluate the effects of meteorological conditions plant growth stage and genetic factors on the yield, quantitative and qualitative composition, on the antioxidant and antimicrobial activities of rosemary essential oil from two Tunisian locations (El Fahs and Matmata) during two successive years. The composition of the essential oils obtained by hydrodistilation from rosemary plants were carried out annually using GC and GC/MS. Results showed the the main constituents were camphor (18.2–28.1%), 1,8-cineole (6.4–18.0%), α-pinene (9.7–13.5%), borneol (4.4–9.5%), and camphene (5.1–8.7%). The principal component and heatmapper analyses showed group segregation of the two studied varities based on major essential oil compounds. Additionally, *in vitro* antimicrobial and antioxidant activities showed that rosemary essential oils had an important ability in scavenging DPPH, as well as a higher bactericidal effect. The seasonal variation, growth stage and genetic pools seemed to be a factors of significant variation of the composition, antimicrobial and the antioxidant activities of the rosemary essential oils. These finding would be taken to use the chemotaxonomy tools to develop a program for Rosmary protection conservation and identification based on essential oil composition.

## Introduction

The *Rosmarinus* L. genus belongs to the Lamiaceae family [1], includes five species in the Mediterranean region: *Rosmarinus officinalis* L., *R*. *eriocalyx* Jourdan and Fourr, *R*. *laxiflorus* (De Noé) Batt., *R*. *la*v*andulaceus* Batt. and *R*. *tomentosus* Huber-Morath and Maire [2]. *Rosmarinus officinalis* (*R*. *officinalis*) is an important ingredient of the "folk pharmacopeia", traditional cuisine, perfumers and cosmetics [3, 4]. The *R*. *officinalis* essential oils showed an antioxidant [5], hepatoprotective [6] and antiulcerogenic effects [7]. Antimicrobial activities against several pathogenic microorganisms of the rosemary essential oil have been shown [8–12]. In addition, rosemary essential oil and extract has received recognition as generally recognized as safe for their intended use, within the meaning of section 409 of the Act Food and Drugs Administratin (FDA, 2014a; FDA, 2014b) and according to the commission Directive 2010/67/EU and commission Directive 2010/69/EU, respectively. *R*. *officinalis* in the mediterranean region is the most exploited species due to its valuable essential oil (EO) [10] and its phenolic content and antioxidant activity [13].

However, rosemary’s oils from natural populations showed high variations in their chemical composition and its efficiency as cosmetics and pharmaceutical ingredients [13]. This non-stability of EO quality, is one of the main causes that this product does not impose itself on the national and international markets. The question is if these variations were mainly correlated to differences in the chemical composition of oils according to the regions [2], the environmental and agronomic conditions [14], the time of harvest [3], the stage of development of plants [15] and the extraction method [16]. Most studies concerned wild population samples showed the genetic diversity within the species [10]. The intraspecific delimitations among taxa remain uncertain because of their high morphological similarities and their high hybridization rate favored by the outcrossing mating system [10]. Pottier-Alapetite [17] recognized for Tunisia one *R*. *officinalis* species including four varieties (four varieties: var. *typicus* Batt., var. *laxiflorus* De Noé, var. *troglodytorum* Maire and var. *lavandulaceum* Batt). Recently, Le floc’h and Boulas [18] grouped all Tunisian taxa into two species. Used isozymic and chemical markers, a distinction between var. *typicus* and var. *troglodytorum* was shown with an intraspecific chemical polymorphism and different chemotypes have been defined in this species according to the dominance of one or more compounds of essential oil [19]. The chemotaxonomy is a plant classification based on chemical constituents [20]. Some studies were conducted to use the chemotaxonomic tools based on essential oils [21, 22].

Considering that, the present work was conducted to evaluate the effects of meteorological conditions (rainfall and temperature), harvesting stage (plant growth stages) in combination with genetic factors on the amount of a secondary metabolite: chemical composition and antioxidant and antimicrobial activities of essential oil of rosemary collected from two different Tunisian regions with different micro-edaphoclimatic environmental.

## Materials and methods

### Plant material

**Fig 1:**
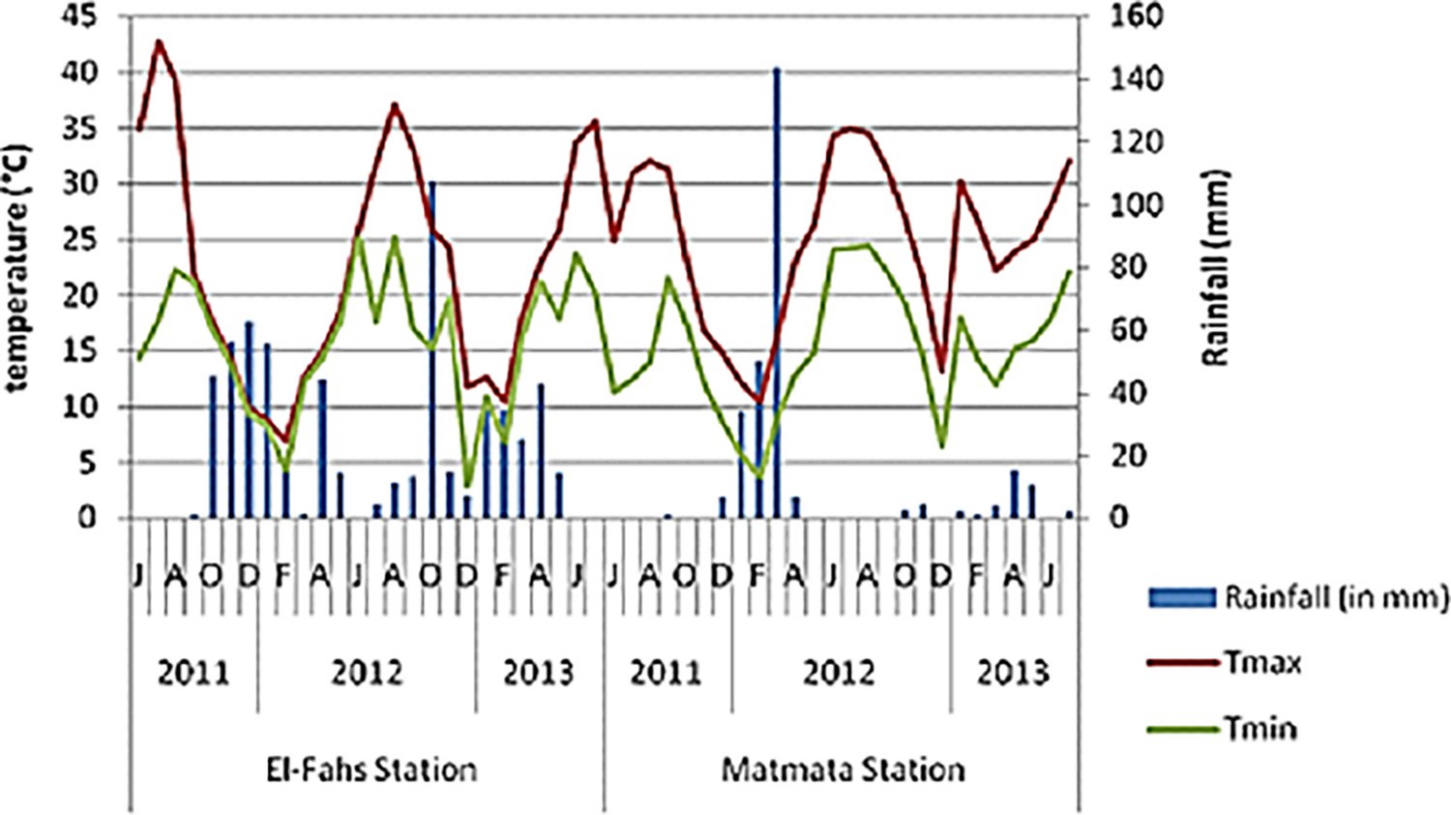
Variations of the pluviometry and the monthly maximal and minimal temperatures of the experimental zones.

**Fig 2:**
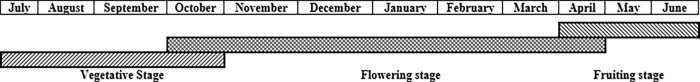
Duration of the different periods of the development stages of *Rosmarinus officinalis* L.

Rosemary samples were collected from two natural populations (Table 1) belonging to different enviromental and edaphic conditions, according to Emberger’s pluviothermic coefficient Q_2_ [23]. Population of *R*. *officinalis* L., var. *troglodytorum*, was collected from the southern (Matmata, upper arid zone) and *R*. *officinalis* L., var. *typicus* from the northern (El-Fahs, sub-humid zone) parts of Tunisia. The meteorological data (the pluviometry and the monthly maximal and minimal temperature) were assessed using weather station placed in each station and presented in Fig 1. These collecting sites have been chosen because they feature rosemary-dominated vegetation, allowing rosemary plants from various climate zones. Five individuals from each population were sampled randomly at over the entire population area at the different stages (vegetative (Vg), flowering (Fl) and fructification (Fr)) during two successives years (June 2011-March 2013) (Vg1, Vg2, Fl1, Fl2, Fr1 and Fr2) (Fig 2) for each region. After that, the fresh vegetable matter was air-dried in well-ventilated room. Vouchers specimens are deposited in the herbarium of the Institute of Arid Lands.

**Table 1:** Location, main ecological traits of the analysed populations of *Rosmarinus officinalis* L.

Variety	Number of population	Locality	Bioclimatic^a^ zone	Latitude	Longitude	Altitude (m)	Rainfall (mm/ year)	Average Temperature (C°)
***troglodytorum***	5	Matmata	Ua	33°32’N	9°58’E	600	100–200	20	
***typicus***	5	El-Fahs	Usa	36°22’N	9°54’E	300	400–500	18.6	

Ua: Upper arid, Usa: sub-humid

^a^ Bioclimatic zones were defined according to Emberger’s (1996) pluviometric coefficient. Q2 = 2000P/(M^2^- m^2^) where P is the mean anuual rainfall (mm)

### Extraction of essential oil

One hundred grams of dried leaves from each sample were submitted to hydro-distillation for 3 h, using a Clevenger-type apparatus. Essential oils were recovred directly and stored in sealed vials protected from light at 4°C until analyses.

### Essential oil analyses

#### Gas chromatography (GC)

The rosemary oil analysis was conducted using A Hewlett-Packard 5890 series II gas chromatograph equipped with HP-5MS capillary column 30 m × 0.25 mm i.d., film thickness 0.25 μm; Hewlett-Packard) and connected to a flame ionization detector (FID). The column temperature was programmed at 50°C for 1 min, then 7°C/min to 250°C, and then left at 250°C for 5 min. The injection port temperature was 240°C and that of the detector 250°C (split ratio: 1/60). The carrier gas was helium (99.995% purity) with a flow rate of 1.2 mL/min and the analysed sample volume was 2 μL. Percentages of the constituents were calculated by electronic integration of FID peak areas, without the use of response factor correction. Mean percentage of compounds in *R*. *officinalis* L. essential oils represented the average calculated on five individuals (n = 5). Retention indices (RI) were calculated for separate compounds relative to (C_7_—C_25_) n-alkanes mixture (Aldrich Library of Chemicals Standards) [24].

#### Gas chromatography/Mass spectrometry (GC/MS)

The isolated volatile compounds were analysed by GC/MS, using an Agilent Technologies 6890 N gas chromatograph. The fused HP-5MS capillary column (the same as that used in the GC/FID analysis) was coupled to an Agilent Technologies 5973B mass-spectrometer (Hewlett-Packard, Palo Alto, CA, USA). The oven temperature was programmed at 50°C for 1 min, then 7°C/min to 250°C, and then left at 250°C for 5 min. The injection port temperature was 250°C and that of the detector was 280°C (split ratio: 1/100). The carrier gas was helium (99.995% purity) with a flow rate of 1.2 mL/min. The mass spectrometer conditions were as follow: ionization voltage, 70 eV; ion source temperature, 150°C; electron ionization mass spectra were acquired over the mass range 50–550 m/z. Identification of the essential oil compounds was based on a comparison of retention indices (RIs) and computer mass spectra library (Wiley 275). The retention indices were determined relative to the retention times for a series of n-alkanes (C_7_—C_25_) [24] using linear interpolation and to those previously reported in the literature [10, 25].

### Antioxidant activity

The antioxidant activity was assessed by 1,1-diphenyl-2-picrylhydrazyl (DPPH) test. The DPPH radical-scavenging activity of essential oils were measured as previously described by Dinis et al. [26] with small modifications. A mixture consisting of 1 mL of methanol and 3 mL of DPPH solution was used as the control. The percentage inhibition of DPPH radical was calculated according to the following formula: % inhibition = [(AB − AA)/AB] * 100, where AB and AA are the absorbance values of the control and of the sample, respectively. The radical-scavenging activity of samples was expressed as the IC50 (μg/mL) reflecting the EO concentration would inhibit 50% of DPPH radical.

### Antibacterial activity

#### Bacterial strains

Antibacterial activities of *R*. *officinalis* L. essential oils were tested against 4 strains of bacteria: two Gram-negative (*Escherichia coli* and *Salmonella typhimurium*) and two Gram-positive (*Bacillus cereus* and *Staphylococcus aureus*).

#### Antimicrobial activity assay

The antimicrobial activity of oils was determined through the disc-diffusion method according to the modified method described by Freney et al. [27]. Briefly, the bacterial suspension used to inoculate Petri dishes had a turbidity of approximately 0.5 McFarland standards. The oils were suspended in DMSO solvent at a concentration of 20 μg/mL. Then, filter paper discs (6 mm diameter) were separately impregnated with 10 μL of the different oils and put on the surface of the inoculated plates (90 mm). The Petri dishes were left at + 4°C for 2 h to facilitate the diffusion of essential oils in agar and then incubated at 37°C for 24 h for bacterial strain. DMSO (10%) was used as a negative control. Gentamicin (10 μg/disk) was used as a positive control. Antimicrobial activity was assessed by measuring the inhibition zone around each disk. All experiments were carried out in triplicate.

### Statistical analysis

The 31 compounds identified of the essential oil was checked by a descriptive statistical analysis using the SPSS software for Windows™ (version 11.5). The percentage of compound were transformed using the arcsine transformation to improve the distribution property. However, this transformation did not yield satisfactory results for 18 variables. Therefore, for compounds having skewed distributions, a nonparametric one-way analysis of variance Kruskal-Wallis test was performed. The chemical population structure was assessed by Linear Discriminant Analysis (LDA). Duncan’s multiple range test (p < 0.05) was used to compare the averages of essential oil yields and the DPPH radical-scavenging activities among populations. Multivariate analyses were used based on essential oil compounds, yield and antoxidant activity including pricipal component analysis (PCA) and heatmap online analysis (http://www.heatmapper.ca/). A heatmap is a graphical representation of data using a color-coding system from lower to highest values. The Rosmary essential oil samples were classifed based on average linkage and using Euclidian distance.

## Results and discussion

### Essential oil yield according to population locations and phenological stages

Sixten spontaneous populations of *R*. *officinalis* populations 1 (var. *troglodytorum*) and populations 2 (var. *typicus*) were collected during the vegetative, flowering and fruiting stages at two years from each studied region. These collecting sites belonged to two different micro-edaphoclimatic environmental conditions. Populations 1 is located at the South West of Tunisia (Matmata) in upper arid climate characterized by a mean rainfall of 100–200 mm/year. The population 2 is localized at the North West of Tunisia (El Fahs) in upper semi-aride climate characterized by a main rainfall of 400–500 mm/year (Fig 1). The monthly means of temperature and precipitation at the sampling locations were shown in Fig 1. The altitudes ranged from 220 m (population 2) to 600 m (population 1) (Table 1). Matmata is drier than El Fahs throughout the year. The vegetations of the collection sites are influenced by the Mediterranean climate, which has less rainfall in the summer. The yield of essential oils at growth stage varied from 1.91% to 3% during 2011–2013 (Table 2). It’s affected by meteorological conditions, years and phenological stage in each region. The average yield of rosemary essential oil was highest in early summer for both sampling locations having a value of 3% in June 2012 (Fr2) for the var. *troglodytorum* collected from the Matmata region and 2.17% in June 2011 (Fr1) for the var. *typicus* from El-Fahs region.

**Table 2:** Yield and chemical composition of the essential oils of *Rosmarinus officinalis* samples.

Station		Matmata region (var. *troglodytarum*)						El-Fahs region (var. *typicus*)						Factor effect (F stat)		
Phenolgical stage (Date)		Vg1	Vg2	Fl1	Fl2	Fr1	Fr2	Vg1	Vg2	Fl1	Fl2	Fr1	Fr2	Station	Date	Station*date
**Yields (%)**		2.25±0.18^b^	2.48±0.23^ab^	2.35±0.20^b^	2.42±0.33^b^	2.42±0.53^ab^	**3.00±0.24** ^**a**^	1.99±0.33^c^	2.06±0.48^c^	1.91±0.29^c^	1.85±0.55^d^	**2.17±0.33** ^**b**^	2.09±0.17^c^	**37.355**	**2.49**	1.26
**DPPH (IC**_**50**_ **en μg/ml)**		2.79 ±0.84	5.62±0.38	2.93±1.21	5.06±0.84	4.74 ±1.49	1.59 ±0.44	4.04 ±1.62	5.32±0.75	4.71±3.03	4.86±0.90	2.94±1.17	3.74±1.00	**2.920**	**5.760**	**3.170**
**Compounds**	**RI**															
tricyclene	925	0.46±0.12	0.36±0.03	0.47±0.05	0.34±0.03	0.34 ±0.07	0.41 ±0.04	-	0.07±0.01	0.11±0.01	0.07±0.01	-	0.08±0.02	**513.41**	**20.12**	**10.82**
**α-pinene**	942	**13.68±0.70**	7.45±1.11	**13.65±0.79**	7.34±0.55	**13.02±0.93**	**14.79±0.75**	**14.03±1.05**	**13.39±1.08**	**13.53±0.94**	**12.56±0.93**	**13.13±1.27**	**12.65±0.47**	**83.797**	**58.796**	**43.818**
**Camphene**	957	**13.35±1.12**	**15.72±1.79**	**13.53±0.65**	**15.19±1.10**	**13.08±1.16**	**13.39±0.93**	3.61±0.40	2.85±0.78	4.66±0.77	2.57±0.27	4.13±0.50	4.30±0.14	**2889.147**	**1.300**	**16.381**
β-Pinene	984	0.31±0.06	1.24±1.07	0.41±0.14	0.72±0.04	0.47±0.22	0.76±0.12	0.97±0.23	-	0.79±0.20	-	1.56±0.25	2.78±0.23	**76.319**	**23.389**	**19.519**
Myrcene	998	0.58±0.07	-	0.57±0.09	-	0.61±0.13	0.79±0.13	0.87±0.09	-	0.82±0.11	-	0.92±0.03	1.37±0.04	**162.037**	**41.244**	**27.152**
α-phellandrene	1011	0.13±0.03	-	0.20±0.14	-	0.13±0.02	0.23±0.05	0.14±0.05	-	0.19±0.06	-	0.16±0.03	0.23±0.06	ns	**3.158**	ns
α-terpipene	1024	0.46±0.09	-	0.93±0.26	-	0.13	0.90±0.15	0.42±0.13	-	0.38±0.10	-	0.53±0.07	0.90±0.04	ns	**ns**	ns
**p-cymene**	1043	**5.80±0.90**	-	**5.11±1.38**	-	**4.72±0.32**	**4.15±0.83**	**4.06±0.18**	-	**4.13±0.29**	-	**3.74±0.17**	-	**13.609**	ns	**4.997**
**1,8-Cineol**	1061	**21.54±2.27**	**37.14±2.48**	**21.63±1.46**	**35.26±1.62**	**23.30±1.57**	**23.26±1.77**	**36.18±1.96**	**53.74±2.46**	**31.64±5.27**	**50.66±1.65**	**30.42±1.84**	**33.78±1.43**	**517.793**	**164.244**	**6.539**
γ-terpinene	1069	0.22±0.06	-	0.40±0.07	-	0.31±0.18	0.64±0.07	0.31±0.15	-	0.24±0.08	-	0.49±0.07	0.58±0.02	ns	**4.196**	ns
α-terpinolene	1095	0.11±0.02	-	0.13±0.04	-	0.14±0.06	0.30±0.06	0.16±0.09	-	0.14±0.04	0.38±0.01	0.28±0.03	0.44±0.03	**26.159**	**44.079**	**6.199**
Linalool	1125	-	-	-	-	-	-	0.62±0.09	-	0.70±0.11	-	0.68±0.14	0.88±0.09	**16.980**	**10.319**	**6.058**
α-Fenchyl alcool	1142	0.02±0.03	-	0.10±0.02	-	0.05±0.00	-	0.14±0.05	-	0.11±0.04	-	0.08±0.01	0.07±0.01	**41.230**	**24.975**	**8.088**
**Camphor**	1178	**28.39±2.07**	**18.88±1.66**	**26.69±1.84**	**18.88±1.02**	**28.72±1.44**	**26.46±1.93**	**19.24±1.91**	**9.00±2.16**	**17.44±1.79**	**9.30±1.40**	**19.15±2.87**	**17.65±1.53**	**547.409**	**95.179**	ns
**Borneol**	1199	4.35±2.06	**8.26±0.94**	4.87±3.05	**8.75±0.76**	4.56±1.59	2.82±0.47	**12.33±1.41**	**10.20±1.55**	**13.46±3.39**	**12.27±1.14**	**14.18±3.24**	**12.32±1.32**	**161.188**	**3.864**	**6.118**
Terpinene-4-ol	1200	1.40±0.60	1.72±0.21	1.18±0.09	1.85±0.15	1.19±0.20	1.10±0.09	0.99±0.11	**5.91±0.19**	0.87±0.01	**5.85±0.27**	-	-	**487.948**	**94.098**	**51.335**
**α-terpineol**	1222	2.72±0.56	-	2.35±0.29	**4.96±0.26**	2.75±0.55	2.67±0.58	**4.33±0.37**	-	**4.71±0.84**	-	**5.30±1.18**	**5.84±0.75**	**97.289**	**10.132**	**34.056**
Actétate de bornyl	1308	1.68±0.87	2.06±0.93	2.33±0.42	1.91±0.51	2.80±0.75	2.69±0.69	0.30±0.07	0.94±0.28	1.06±0.24	1.67±0.51	1.07±0.36	1.14±0.24	**91.613**	**3.671**	ns
Carvacrol	1320	0.22±0.04	0.62±0.24	0.22±0.06	0.57±0.23	0.07±0.02	0.14±0.07	0.12±0.05	0.41±0.16	0.19±0.02	0.45±0.11	0.11±0.04	0.14±0.02	**9.192**	**25.147**	**3.389**
Eugnol	1376	0.11±0.02	0.31±0.01	0.12±0.02	0.33±0.06	0.09±0.03	0.12±0.02	0.10±0.02	-	0.16±0.04	-	0.24±0.12	0.16±0.06	**4.339**	**1.960**	**5.134**
α-copaene	1383	0.39±0.35	0.26±0.04	0.11±0.02	0.51±0.17	0.04±0.01	0.08±0.02	-	0.24±0.00	0.09±0.02	0.27±0.05	0.07±0.02	0.11±0.03	ns	**4.724**	**0.357**
Methyl eugnol	1422	0.52±0.12	0.80±0.18	0.59±0.07	0.83±0.12	0.56±0.17	0.69±0.20	0.10±0.03	-	0.12±0.03	-	0.15±0.06	0.15±0.02	**129.055**	**10.494**	ns
**β-caryophyllene**	1433	0.24±0.08	0.42±0.06	0.49±0.22	0.57±0.21	0.23±0.14	0.48±0.14	0.54±0.08	0.92±0.11	**1.54±0.41**	**1.33±0.27**	**1.35±0.31**	**1.72±0.37**	**148.449**	**7.738**	**5.513**
α-Humulene	1465	0.11 ±0.00	-	0.09±0.02	-	0.04±0.01	0.07±0.02	0.07±0.00	0.20±0.06	0.20±0.04	0.30±0.10	0.13±0.03	0.25±0.03	**20.546**	**4.629**	**4.761**
α-amorphene	1485	0.18±0.13	0.29±0.01	0.16±0.08	-	0.04±0.01	0.09±0.05	0.05±0.00	0.07±0.00	0.11±0.02	0.52±0.06	0.07±0.03	0.13±0.02	ns	**4.061**	**2.376**
δ-Cadinene	1532	0.09±0.03	0.32± 0.01	0.25±0.05	0.29±0.11	0.05±0.02	0.15±0.08	0.14±0.00	0.30±0.18	0.24±0.06	0.37±0.14	0.14±0.06	0.24±0.08	**7.089**	**10.063**	ns
Caryophyllene oxid	1610	0.12±0.03	0.16± 0.01	-	-	0.18±0.09	-	0.24±0.04	0.17±0.14	0.33±0.07	0.53±0.00	0.31±0.07	0.35±0.04	**15.291**	**6.723**	**3.285**
gamma-Eudesmol	1640	0.40±0.13	-	0.52±0.16	-	0.31±0.05	0.42±0.17	0.28±0.05	-	0.49±0.10	-	0.33±0.07	0.26±0.12	ns	**4.629**	ns
α-Elemene	1659	0.18±0.02	-	0.24±0.04	0.32±0.07	0.08±0.00	0.20±0.08	0.10±0.01	-	0.21±0.05	-	0.18±0.05	0.22±0.05	ns	**2.945**	ns
α-Eudesmol	1676	2.15±1.30	1.81±0.58	2.11±0.30	1.77±0.72	1.41±0.29	1.93±0.87	0.49±0.12	-	0.77±0.16	-	0.61±0.19	0.41±0.16	**36.469**	ns	**2.213**
**All identified components (%)**		**99.6**	**99.6**	**99.5**	**98.9**	**99.7**	**97.36**	**99.5**	**99.2**	**93.1**	**99.91**	**99.18**	**98.18**			
**Monoterpene hydrocarbons (%)**		**29.3**	**24.77**	**35.40**	**23.59**	**32.95**	**36.36**	**24.57**	**16.31**	**24.99**	**15.58**	**24.94**	**23.33**			
**Oxygenated monoterpenes (%)**		**58.64**	**66.62**	**57.04**	**70.27**	**60.64**	**56.45**	**74.03**	**79.26**	**69.12**	**78.53**	**69.91**	**70.68**			
**Monoterpene dioxygenated (%)**		**2.31**	**3.17**	**3.04**	**3.07**	**3.45**	**3.50**	**0.50**	**0.94**	**1.34**	**1.67**	**1.46**	**1.45**			
**Sesquiterpene hydrocarbons (%)**		**1.19**	**1.29**	**1.34**	**1.69**	**0.48**	**1.07**	**0.90**	**1.73**	**2.39**	**2.79**	**1.81**	**2.67**			
**Sesquiterpene oxygenated (%)**		**2.67**	**1.97**	**2.63**	**1.77**	**1.90**	**2.35**	**1.01**	**0.17**	**1.59**	**0.53**	**1.25**	**1.02**			

Components are listed in order of elution in apolar column (HP-5). RI: retention indices calculated using an polar column (HP Innowax). Compound proportions were calculated from the chromatograms obtained on the HP Innowax column. Values are given as mean ± SD (n = 3). ns: not significant, Tr : trace, - : not determined; Vg, Fl., Fr: vegetative, flowering and fructifying stages.

The var. *troglodytarum* from Matmata region showed the highest EO yield at the fuiting stage from April to June distinguished by a decrease in precipitation, since the average precipitation was 6.8 mm in April 2012, absent in May 2012 and in June 2012 and the average temperature was around 25°C.

The highest EO yield in fruiting phase can be explained by the lower average precipitation Sotomayor et al. [28] analysed the effect of water level on the quality of essential oil in the thymol chemotype of *Thymus zygis* subsp. *gracilis* and established that the highest amount of essential oil was produced under the lowest (30%) level of watering. It has been reported that lower amounts of moisture increase the yield of essential oil in phenolic chemotypes of other species from Lamiaceae family: for example, the carvacrol chemotype of wild oregano (*Origanum vulgare*) accumulated more essential oil under lower rainfall [29].

On the other hand, June 2011 in both sampling locations, coincided with the highest temperature compared to other years. Temperature can influence the accumulation of essential oil in different bearing plants both positively and negatively [30]. Analogous results were also obtained through investigations on other species of the Lamiaceae family. The essential oil yield was negatively correlated with higher temperature in wild *Feoniculum vulgare* from Iran [31], and positively correlated in a *Eucalyptus* species from Brazil [32]. The phenolic (thymol and carvacrol) chemotypes of *T*. *vulgaris* growing in natural habitats occur predominantly at hot and dry sites [33, 34].

In conclusion, whatever the phenological stage, the *troglodytorum* var. from Matamta station showed the highest EO yield particularly in the fruiting phase. This highest yield was recorded also for the same variety *troglodytorum* compared to other Tunisian varities studied by Zaouli et al. [10].

### Variation of essential oil composition according to population locations and phenological stages

The EO composition was mainly investigated using both GC and GC/MS techniques and the percentages of the identified compounds were listed in Table 2. Thirty-one compounds representing 93.1 to 99.91% of the total essential oil were identified At all stages, monoterpenes (hydrocarbons and oxygenated) were dominant in *R*. *officinalis* EO independently to the environmental conditions These monoterpenes, represented mainly by 1,8-cineole (33.42–53.7%), α-pinene (12.65–14.03%) and borneol (10–14.18%) in *typicus* variety and by 1,8-cineole (21.54–37.14%), camphor (18.88–28.72%), camphene (13.08–15.72%) and α-pinene (7.34–14.8%) in *troglodytorum* variety. Based on dominant components of the essential oils, the *R*. *officinalis* is characterized as plants with an intraspecific chemical polymorphism. Different chemotypes have been defined in the *R*. *officinalis* varieties according to the dominance of one or more compounds of essential oil. Two chemotypes are detected composed by the chemotype 1, dominated by 1,8-cineole/camphor including the samples from Matmata (*troglodytorum* variety) and the chemotype 2, dominated by 1,8-cineole including those from El Fahs (var. *typicus)*. Recent studies of rosemary essential oil composition of indigenous and cultivated plants in the Mediterranean area revealed the existence of 6 monodominant and 6 intermediate chemotypes. The most recorded monodominant are 1,8- cineole and camphor chemotypes. Less common are verbenone, and α-pinene chemotypes, while in only one sample linalool and *p*-cymene chemotypes were recorded. Intermediate chemotypes charcaterised by 1,8- cineole/linalool, 1,8-cineole/camphor and 1,8-cineole/camphor/borneol were also recorded for only single sample [35].

Referring to obtained results (Fig 1 and Table 2) we noted that the higest amounts of 1,8-cineole were accumulated at vegetative and floraison stages in the two *R*. *officinalis* varieties. While the camphor concentration was higher at vegetative and fruiting stages. Variation in the essential oil quantity and the predominant compounds at different phenological stages is a characteristic of essential oil-bearing plants [36].

During all growth stages the same genotype can synthesize oils with different composition. This criterion would be used to define different chemotypes confirms the opinion that the essential oils composition depends on the time of collect. Also, for the definition of chemotypes it is not enough to base this on a chemical analysis of oil from one phenophase only [35].

On the other hand, in El Fahs region, the content of 1.8-cineole in EOs of var. *typicus* was significantly higher compared to *troglodytorum* variety. At all development stages the content of camphor was higher in *troglodytorum* compared to *typicus* variety EO. The difference not only in the major compounds but also in the minor compounds.

In addition, the results also showed that the percentage of camphor and α-pinene have significantly decreased, sometimes up to half during the second years. An increase in the percentage of 1.8 cineol, borneol and camphene, was revealed at Vg2 and Fl2. The disappearance of some compounds such as myrcene, p-cymene, alpha-phellandrene, ɣ-Terpinene and α-terpinolene at Vg2 and Fl2 in both varieties was also noted (Table 2). It has been noted that the icrease of some compounds amount in essential oils are correlated by changes in the amounts of other compounds [34, 37]. These significant differences between chemical compositions of EO in the two studied varieties can be attributed to genetic, and the geographic origin factors. Indeed, geographical, climatic and pedological characteristics of habitats explained the significant variation of EO from studied samples The geographical distribution of different chemotypes of *R*. *officinalis* essential oils are largely due to the environmental characteristics and the stages of ontogenesis as revisousely reported [35]. Vaiciulyte et al. [36] added that photosynthetically, active solar radiation and sunshine duration affect the amount of essential oil and major compounds of *Thymus pulegioides*.

Previous studies on the chemical composition of rosemary oil showed that the main components were camphor/1,8-cineole/α-pinene, and the intraspecific chemical variability between plants belonging to different geographical areas was noted [38–41]. Our results are in agreement with previous study carried out on rosemary species collected from Spain and characterized by 1,8-cineol and camphor as dominant compounds [42]. Likewise, these two compounds have also been described as the most dominant components in essential oils of Tunisian rosemary species [10]. On the other hand, Ojeda-Sana et al. [43] determined that rosemary essential oil collected from Argentina have α-pinene or myrcene as the main compound. The present results, showed that the geographical origin, altitude and seasons would be a source of variation the composition of rosemary essential oil composition as noted previsoulely on the quality and quantity of Iranian rosemary EO characterized by the 1.8-cineole (5.32–28.29%), camphor (1.58–25.32%) and α-pinene (14.19–21.43%) as the main constituents. Iranian accessions also exhibited chemical variability for other major compounds such as borneol, camphene, bornyl acetate. In addition, authors founded a positive and negative correlations between major constituents and environmental factors.

If we consider the same genetic origin and the micro-edaphoclimatic environmental conditions, we noted that the annual differences of meteorological conditions were the main source of variation in yield of the essential oils and/or of their major compounds. Therefore, the meteorological conditions including rainfall, temperature can influence on EOs and theirs major compounds in *R*. *officinalis*. With this possible variation the necessity of specifying the composition of rosemary essential oil according to its geographical origin and harvest date is a crucial step to conduct biological activities the essential oils.

### Biological activities

#### DPPH free radical scavenging assay

The concentration of major compounds in rosemary EO showed seasonal variation and a significant relationship with precipitation and temperature in each sampling locations. The seasonal variation affected the chemical composition of EOs and could influence the antioxidant and antibacterial activities.

**Fig 3:**
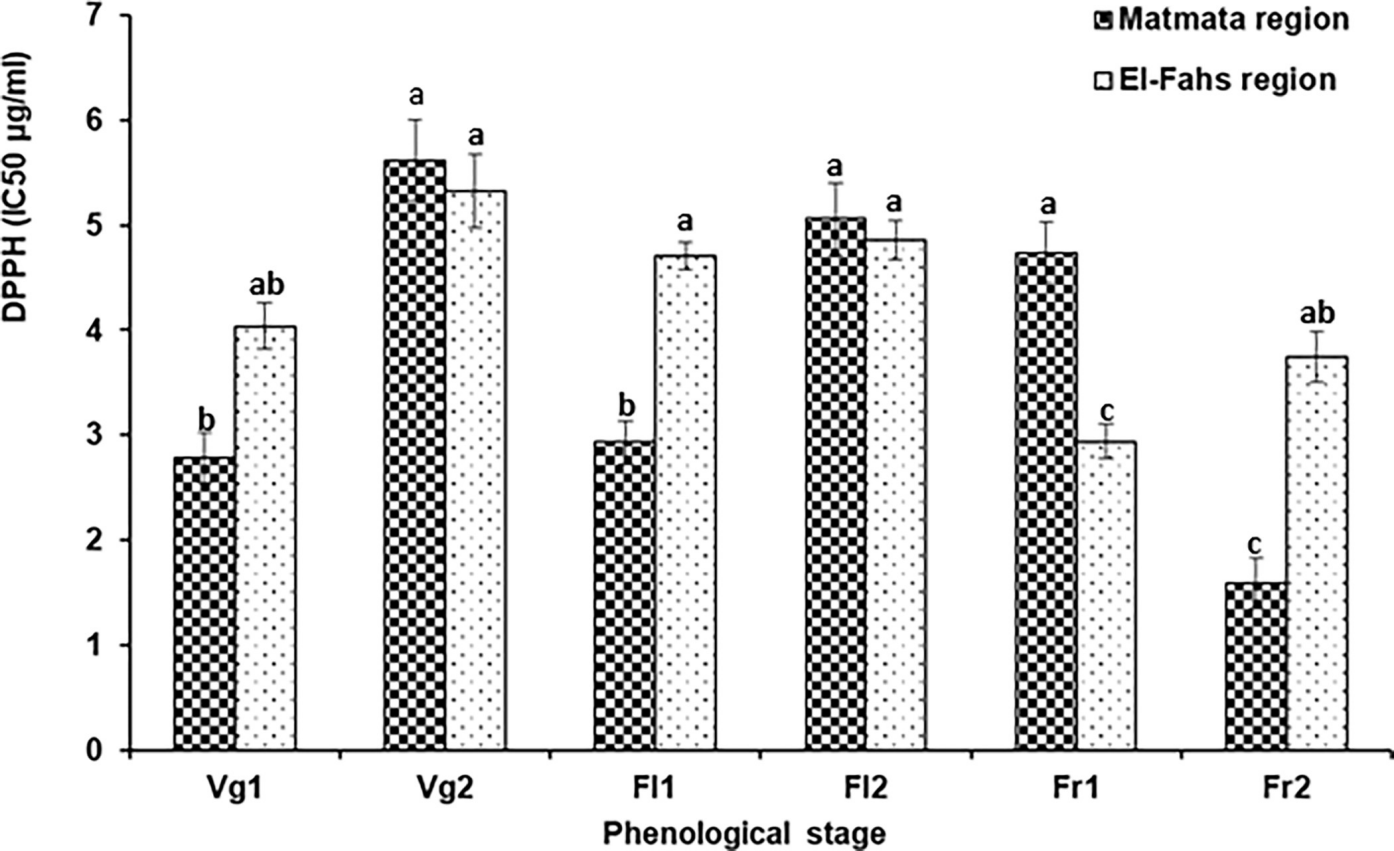
Antioxidant activity of the essential oils of *Rosmarinus officinalis* samples.

The antioxidant activity of EOs rosemary collected from the two sampling locations at differents phenologic stage (Fig 2) for two years were evaluated by DPPH (Fig 3). Significant variations (P < 0.05) were observed in antioxidant activities of rosemary EO according to the geographic origin and seasonal variations. Based on DPPH assay, the determined values ranged from 2.94 to 5.32 μg/mL for the oils extracted from El-Fahs and between 1.59 and 5.62 μg/mL from Matmata. Rosemary EO is well known by its high antioxidant capacity [44]. The highest antiradical activity of rosemary essential oils was detected at Matmata area (var. *troglodytorum)* (IC_50_ average = 1.59 μg/ mL) at Fr2 (June 2012, early summer), followed by the OE extracted at Vg1 (October 2012) (IC_50_ average = 2.79 μg/ mL). From the upper semi-arid of var. *typicus* (El-Fahs), the best activity was detected in EOs extracted during the post-flowering stage (Fr1) (IC_50_ average = 2.94 μg/mL). In addition, the low antioxidant activities of EOs from the two collecting regions were recorded at Vg2 stage (IC_50_ average = 5.62 μg/mL for *troglodytorum* variety and IC_50_ average = 5.32 μg/mL for *typicus* variety) (Fig 3). The antiradicalaire activity of *R*. *officinalis* essential oil *R*. *officinalis* had a positive relationship with aridity. According to Table 2 and the Fig 1, the upper arid (Ua) region includes the *R*. *officinalis* var. *troglodytorum* characterized by a high antioxidant activity compared to the upper semi arid (Usa) region includes the *R*. *officinalis* var. *typicus*. Matmata has a hot climate, while El Fahs has a moderately hot climate. Theses results corroborate with other studies [10]. It could be deduced also that the difference observed in the antioxidant activity level of the two varieties may be due to the variation of the two major compounds contents including the 1.8-cineole and camphor. Indeed, the antioxidant activity increased with camphor and conversely with 1.8-cineole. Compared to other native Mediterranean plants, rosemary can withstand prolonged drought by avoiding damage to its photosynthetic organs. Seasonal variation is associated with certain changes in soil moisture and temperature, which may lead to variations in the biosynthetic pathways of primary and secondary metabolites [45]. The maximum trapping capacity of the oils collected during the flowering phase for the two sampling locations could be explained by the richness of EO in 1.8 cineol, camphene, borneol and camphor compounds [46]. Although camphor and 1,8 cineole were reported as the principal antioxidant in rosemary EO [47] and, also, other compounds as the α-pinene, β-pinene, and 1,8-cineole compounds [48]. It has been noted a correlation between antioxidant components including 1,8-cineole, α-pinene, camphor, borneol, and transcaryophyllene and the antioxidant activity [48]. Various factors such as environmental factors, sampling techniques, extraction methods, plant organs and geographic origin could affect the amount of biomolecules responsible for the antioxidant capacity [49].

### Antimicrobial activity

Antimicrobial activity of rosmery essential oils was tested against four common bacteria pathogens Results showed that the rosmary essential oils exhibited efficient effect against all tested microorganisms (Table 3). The *R*. *officinalis* L. is an important medicinal plant and its essential oil characterized by noteworthy antimicrobial activity [37, 39, 50].

**Table 3:** Antibacterial activity of *Rosmarinus officinalis* essential oil samples.

Oils (10 μL/disc)														Gentamycin (10 μg/disk /disc)
Mean inhibition diameter (mm)														
Bercteria	Source N°	Matmata (var. *troglodytorum*)						El-Fahs (var. *typicus*)						
		Vg1	Vg2	Fl1	Fl2	Fr1	Fr2	Vg1	Vg2	Fl1	Fl2	Fr1	Fr2	
***S*. *aureus***	*ATCC 6538*	24.15±2.91^a^	17.90±1.52^bc^	25,80±2.96^a^	14.80±2.16^cd^	12.5±1.80^d^	19.20±2.64^b^	14.88±2.15^b^	19.29±1.86^a^	15.11±2.54^b^	14.43±1.76^bc^	12.33±1.67^cd^	11.60±1.81c^d^	21±0,5
***S*. *typhimurium***	*NRLB 4420*	23.40±2.60^a^	18.30±1.64^a^	23.50±1.60^a^	19.40±1.52^a^	9.20±1.12^b^	21.090±4.92^a^	14.79±3.23^bc^	16.57±1.71^ab^	14.00±0.80^cd^	17.71±1.27^a^	12.40±1.44^c^	12.21±0.99^c^	22±0,2
***B*. *cereus***	*ATCC 11778*	22.70±2.84^a^	18.80±2.04^ab^	20.70±1.64^ab^	17.40±1.04^b^	11±1.80^c^	19.50±2.40^ab^	18.82±4.41^b^	19.14±1.59^a^	15.27±2.19^bc^	17.86±1.31^ab^	17.87±3.36^ab^	14.30±3.21^c^	20±0
***E*. *coli***	*ATCC 10536*	28.05±2.24^a^	17.70±1.70^b^	26.20±3.64^a^	16.20±1.04^b^	11.10±1.72^c^	27.60±2.12^a^	20.95±4.64^a^	19.36±1.64^ab^	17.20±3.41^bc^	18.64±0.88^abc^	12.93±1.47^d^	15.93±3.15^c^	18±0

Vg, Fl., Fr: vegetative, flowering and fructifying stages.

Values are given as mean ± SD (n = 3). For the same region and bacteria, means followed by the same letter did not share significant differences at p < 5% (Duncan test).

The rosemary samples from Matmata showed the against *E*. *coli* than those from El Fahs at Vg1 (Sep 2011). This activity against *E*. *coli* with 28.05 mm as inhibition zone diameter was superior to those previously reported of *R*. *officinalis* EO from Tunisia (18.17 mm) [10] and from Iran (17 mm) [9]. It has been demonstrated that hydrocarbons and oxygenated monoterpenes in the essential oils are able to destroy cellular integrity, and thereby inhibit respiration and ion transport processes. This is strongly supported by the effects of different essential oils components on outer membrane permeability in Gram-negative bacteria. Most studies investigating on the action of essential oils against food spoilage organisms and food borne pathogens agree that, essential oils are slightly more active against Gram-positive than Gram-negative bacteria [51].

The variation of the antimicrobial activitiy between the all investigated essential oils samples can be attributed to their chemical composition, in particular to their abundant compounds including the 1.8-cineole, camphor, and camphene. The same suggestion was mentioned by Zaouali et al. [10] of two varieties of Tunisian rosemary (*typicus* and *troglodytorum*). However, other authors confirm that the antimicrobial effect of rosemary can not be explained only by the presence of a single substance in large amounts, but by the synergy of several components in smaller amounts [38, 42, 43, 49].

The rosemary EO sampled in this study showed variations in chemical composition mainly in their major compounds, suggesting the variation of thier biological activities (antioxidants and antimicrobial) according the season. Therefore, the antioxidants and antimicrobial potential of rosemary EO changed during the sampling period. The changes in chemical composition and the biological activities during different seasons have been previousely reported [3, 29, 45].

### Principal component analysis

**Fig 4:**
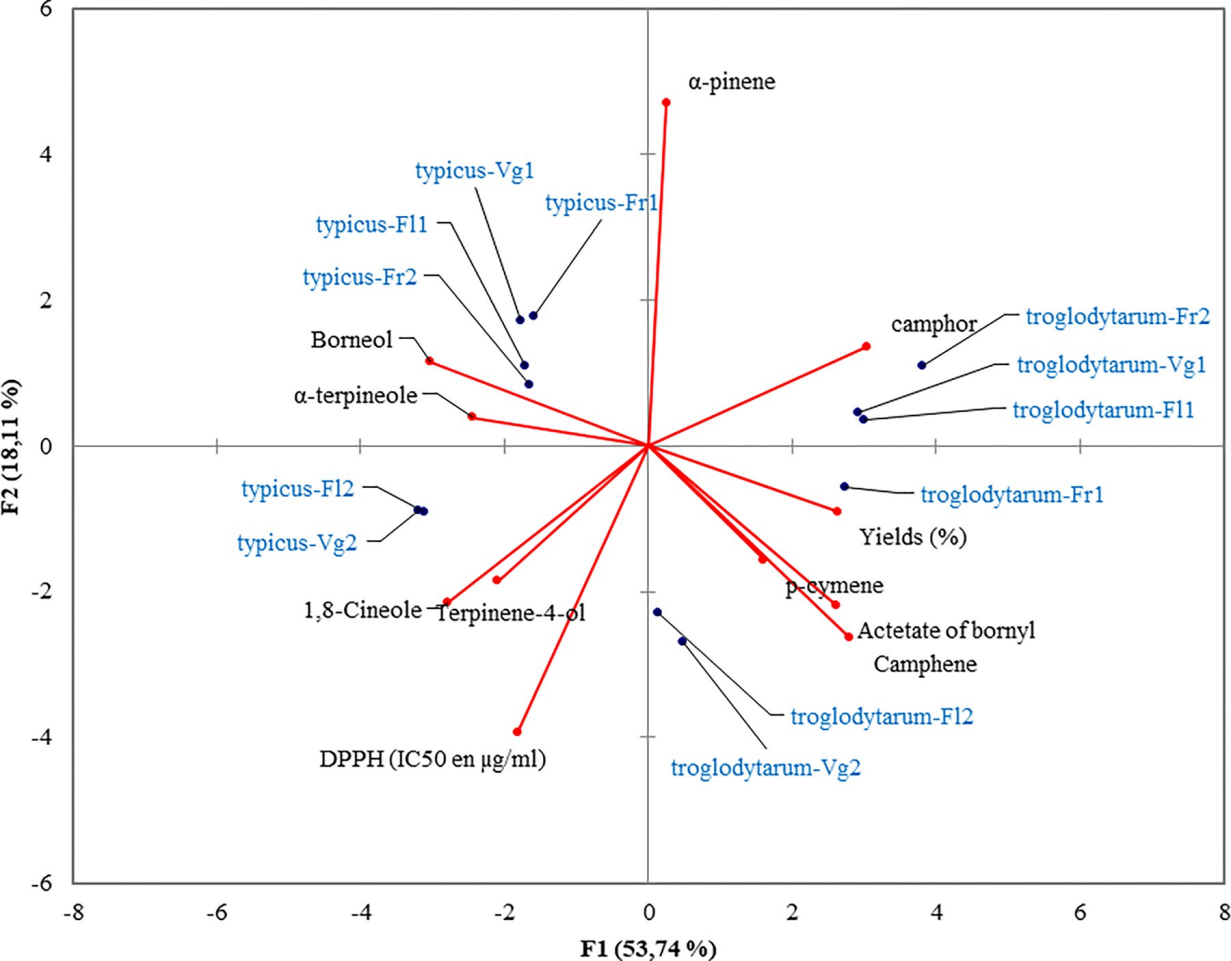
Principal component analysis plot of Rosmary samples and essential oil compounds.

The principal component analysis (PCA) was applied using the essential oils data. This method establishes mathematical criteria that allow similarities between samples or clusters to be expressed quantitatively. The PCA plot showed the segregation of the two varieties, *typicus* and *troglodytorum* (Fig 4). The two first principal component expressed 71,85% of total variation. The variety *typicus* was divided on two groups according to the growth stage and growing season. All plant growth stages for the first season (Vg1, Fl1, Fr1) and the fruiting stage of second season constituted one group. This groupe was formed based on their content on broneol and α-terpineol. The *typicus* variety from two growth stages (Fl2 and Fr2) of the second season were grouped based on their content of 1,8-cineole and terpinen-4-ol. The second groups were formed by *troglodytorum* variety at all growth stages in two the seasons (Fig 4 and Table 4).

**Table 4:** Codes used in principal component and heatmapper analyses.

	Stage	Fig code
troglodytarum-Vg1	Vegetative stage from the first season (Vg1) and the second season (Vg2)	1
troglodytarum-Vg2		2
troglodytarum-Fl1	Flowering stage from the first season (Fl1) and the second season (Fl2)	3
troglodytarum-Fl2		4
troglodytarum-Fr1	Fruiting stage from the first season (Fr1) and the second season (Fr2)	5
troglodytarum-Fr2		6
typicus-Vg1	Vegetative stage from the first season (Vg1) and the second season (Vg2)	7
typicus-Vg2		8
typicus-Fl1	Flowering stage from the first season (Fl1) and the second season (Fl2)	9
typicus-Fl2		10
typicus-Fr1	Fruiting stage from the first season (Fr1) and the second season (Fr2)	11
typicus-Fr2		12

**Fig 5:**
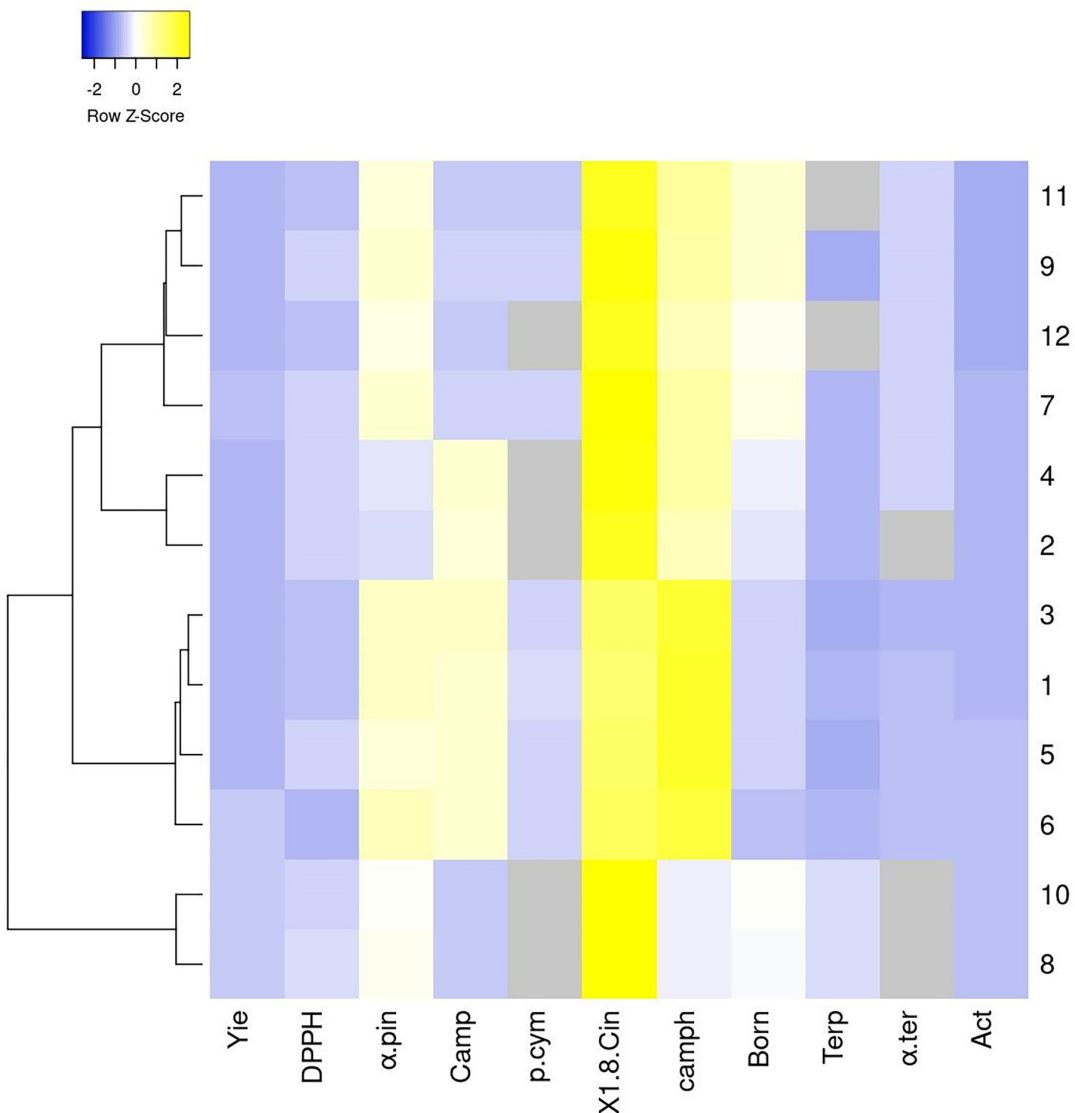
Heatmap plot of Rosmary samples and major essential oil compounds using color score.

Heatmapper analysis showed the same groups obtain by PCA (Fig 5 and Table 4). The color reflects highest (yellow) and lowest (blue) values using color score as shown in Fig 5. The *troglodytorum* (2 and 4) and *typicus* (10 and 8) varieties were closely at vegetative and flowering stage from second seasons. The 1,8-cineole, α-pinene, camphene and camphor compounds were the highest compound values having yellow color.

## Conclusion

The genetic, locality, developement stages and seasons influenced significantly the composition, antimicrobial and antioxidant activities of studied rosemary essential oils. Rosemary EO antioxidants and antibacterial activities were correlated with major compounds.

Based on the relative concentrations of the major components in rosemary oils, the multivariate analyses including PCA and heatmapper analyses, two chemotypes were defined. This finding would be used as a criterion for selecting the season and harvest area of rosmery to extract essential oils having crucial potentialities.

## Supporting information

S1 Data (XLSX)
